# Supplementary material for: HDLBP binds ER-targeted mRNAs by multivalent interactions to promote protein synthesis of transmembrane and secreted proteins
Source: Nat Commun. 2022 May 18;13:2727. doi: 10.1038/s41467-022-30322-7 (PMC9117268; doi:10.1038/s41467-022-30322-7)
Supplement: Supplementary file 1 — Supplementary Information [file 41467_2022_30322_MOESM1_ESM.pdf]

Supplementary Information for

HDLBP binds ER-targeted mRNAs by multivalent interactions to promote protein synthesis of transmembrane and secreted proteins

Ulrike Zinnall[1,+], Miha Milek[1,2,+,*], Igor Minia[1], Carlos H. Vieira-Vieira[1], Simon Müller[3], Guido Mastrobuoni[1], Orsalia-Georgia Hazapis[1], Simone Del Giudice[1], David Schwefel[4], Nadine Bley[3], Franka Voigt[5], Jeffrey A. Chao[5], Stefan Kempa[1], Stefan Hüttelmaier[3], Matthias Selbach[1,4], Markus Landthaler[1,6,*]

Content:

Supplementary Figure 1-7

Supplementary Table 1

Supplementary Figure 1

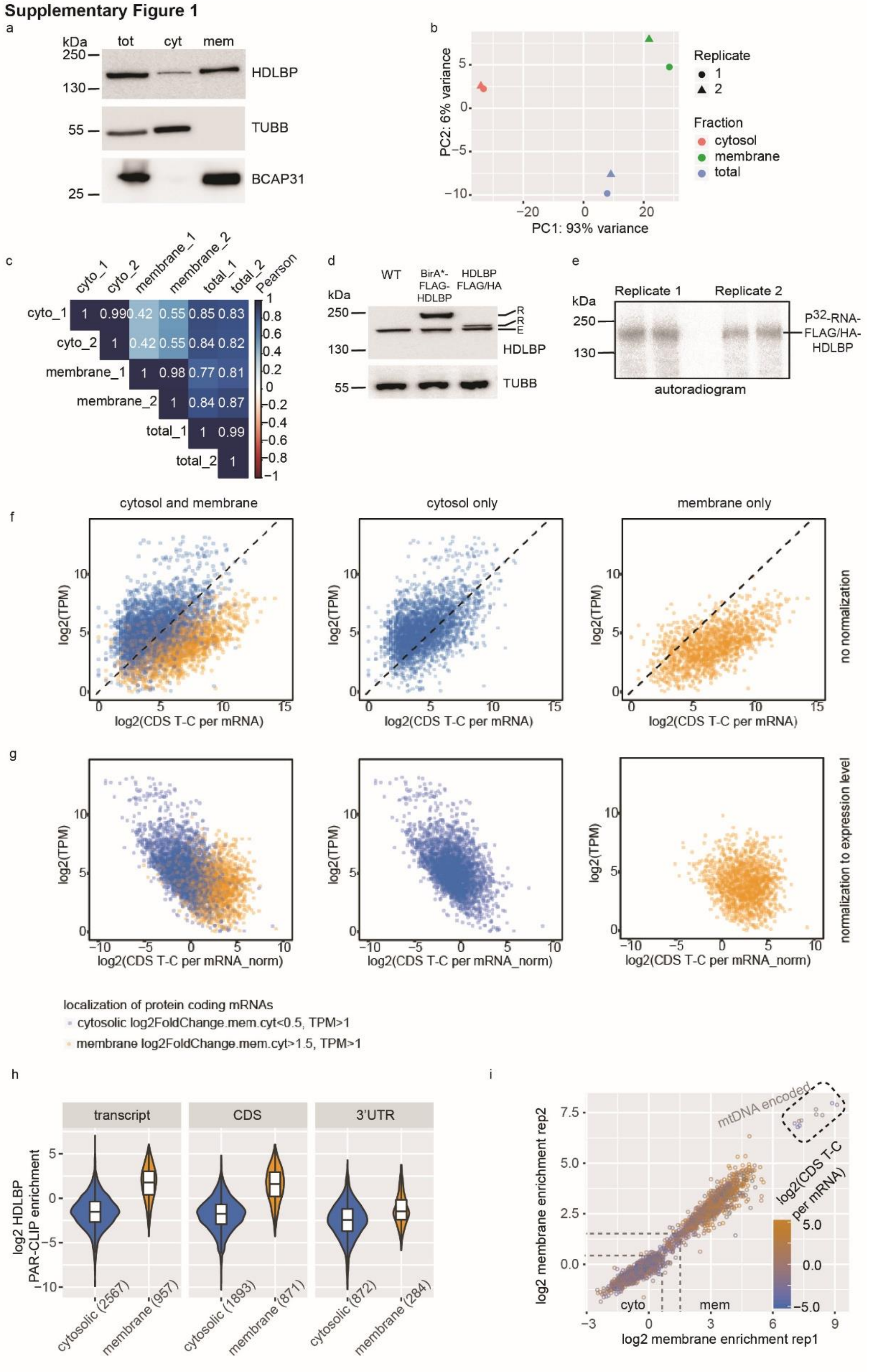

Supplementary Figure 1. HDLBP UV-crosslinks to mRNAs in CDS and 3'UTRs

(a) Western analysis of sub-cellular fraction purity using marker proteins (ER: BCAP31, cytosol: TUBB, both compartments: HDLBP). Membrane-bound fraction contained ER and mitochondria.

(b) Principal component analysis using variance stabilized transformation of mRNA-seq read counts per gene.

(c) Correlation matrix of pairwise comparisons between non-transformed mRNA-seq read counts per gene derived from different fractions. Pearson coefficients are shown.

(d) Western analysis of HEK293 cells stably expressing FLAG/HA-HDLBP or BirA*-FLAG-HDLBP.

(e) Autoradiogram of immunoprecipitated, radio-labeled and SDS-PAGE separated HDLBP-RNA complexes. Migration of the band corresponding to P^{32}-RNA-FLAG/HA-HDLBP is indicated.

(f) Scatter plots of non-normalized HDLBP crosslink signal in the coding sequence versus mRNA expression level (total fraction). mRNAs were classified according to their localization determined by fractionation in combination with mRNA-seq (cytosolic or membrane-bound).

(g) Scatter plots of expression-normalized HDLBP crosslinking signal in the coding sequence versus mRNA expression level (total fraction). mRNAs were classified according to their localization determined by fractionation in combination with RNA-seq (cytosolic or membrane-bound).

(h) Comparison of the distributions of log2-transformed HDLBP PAR-CLIP enrichment computed for whole transcript (5'UTR, CDS and 3'UTR), coding CDS and 3'UTR crosslink signals. Sizes of groups (n) are indicated as absolute numbers. Lower and upper hinges of box plots correspond to the 25th and 75th percentiles, respectively. Upper and lower whiskers extend from the hinge to the largest or smallest value no further than 1.5 * interquartile range from the hinge, respectively. Center lines of box plots depict the median values.

(i) Scatter plot of mRNA-seq log2-transformed membrane enrichment in 2 biological replicates. Data points are coloured according to the log2-transformed HDLBP PAR-CLIP crosslinking signal in coding sequences (CDS). mRNAs with TPM at least 10 were included in this analysis.

(a – i) Source data are provided as a Source Data file.

Supplementary Figure 2

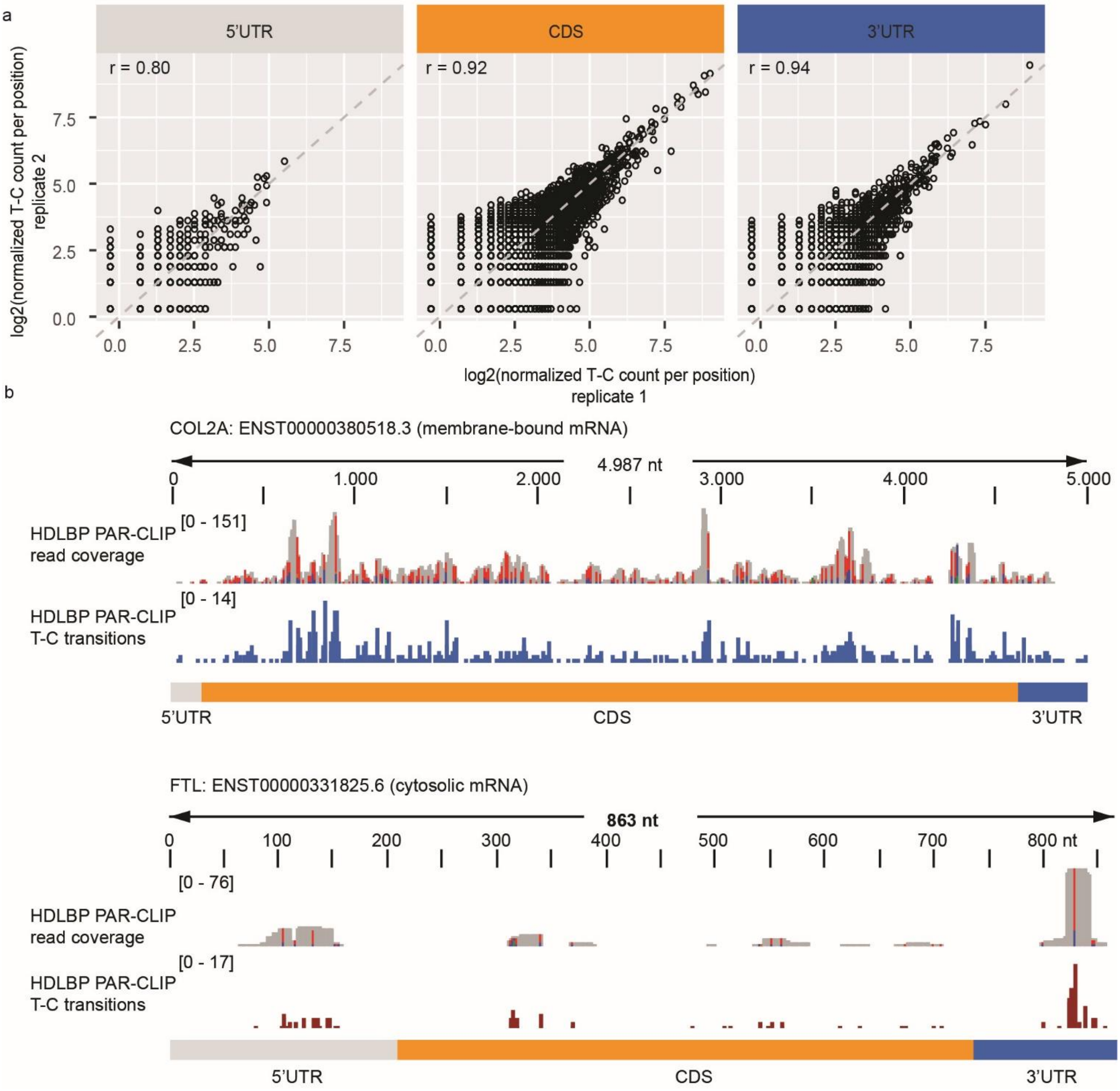

Supplementary Figure 2. HDLBP PAR-CLIP reproducibility and specific mRNA contacts

(a) Reproducibility of T-C transitions between replicates. Scatter plot of normalized T-C transitions per million for 2 HDLBP PAR-CLIP replicates. Pearson correlation coefficients are given.

(b) Browser representation of PAR-CLIP read coverage and T-C transitions in COL2A1 (membrane-bound) and FTL (cytosolic) mRNAs. Reads were mapped to human mRNA sequences. 5'UTR, CDS and 3'UTR regions are indicated. Transcript IDs are indicated.

(a, b) Source data are provided as a Source Data file.

Supplementary Figure 3

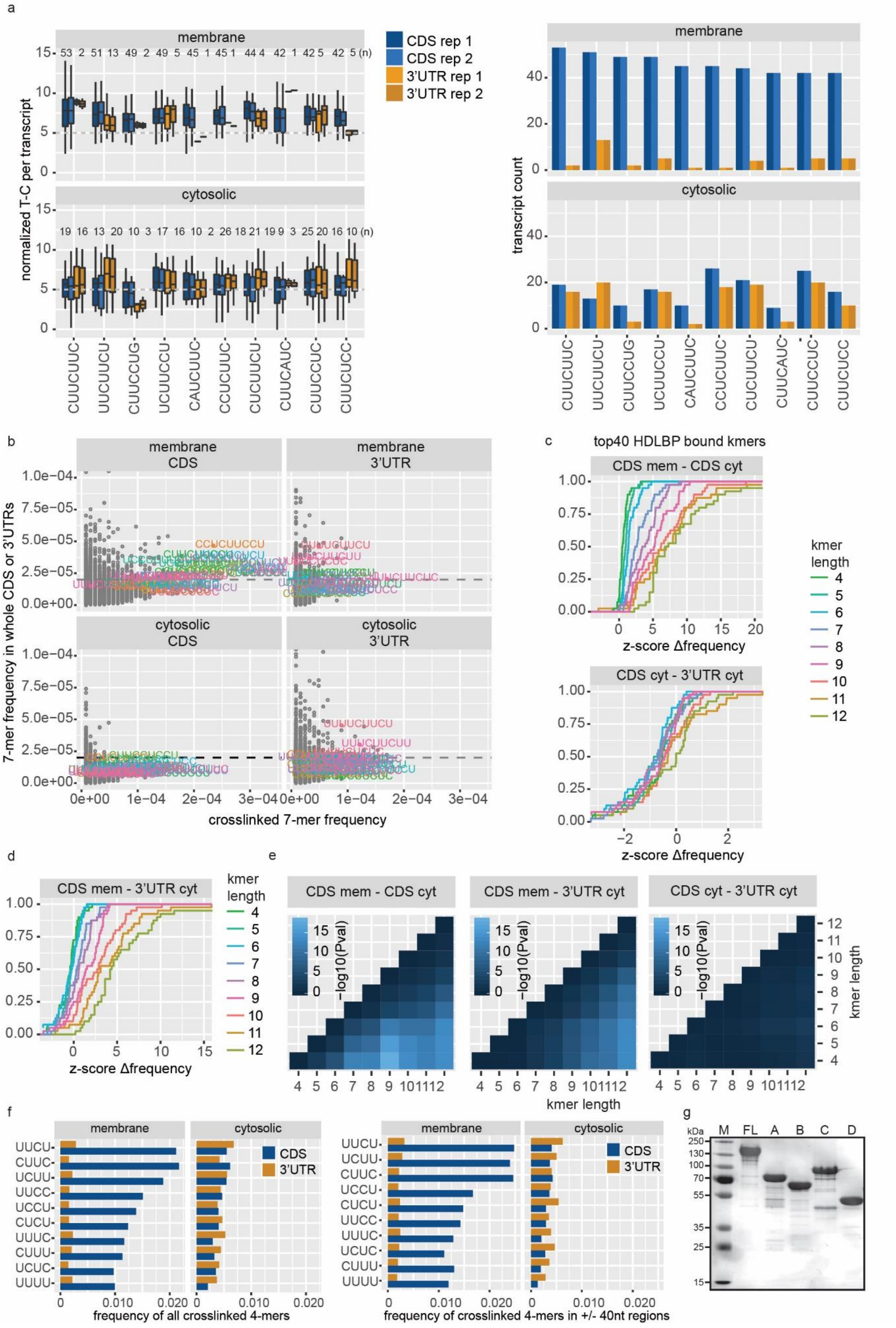

Supplementary Figure 3. HDLBP interacts with CU-rich RNA sequences

(a) Signal of HDLBP crosslinked 7-mers. T-C transition counts for all possible 7-mers within a CDS and 3'UTR were summed for each transcript and its regions, followed by normalization to the length of the respective CDS/3'UTR and to the transcript expression level. The 7-mers were then ranked according to the median of the log2-transformed normalized crosslinked k-mer signal. Top 10 7-mers are shown for both PAR-CLIP replicates (left). Absolute number of transcripts in each class shown in the left panel are given above the box plots and in the right panel. Lower and upper hinges of box plots correspond to the 25th and 75th percentiles, respectively. Upper and lower whiskers extend from the hinge to the largest or smallest value no further than 1.5 * interquartile range from the hinge, respectively. Center lines of box plots depict the median values.

(b) Comparison of the HDLBP crosslinked 7-mer frequency to the corresponding frequency in the transcript region (CDS or 3'UTR). Top 20 crosslinked 7-mers are shown in color.

(c) Distribution of z-scores calculated from differences in the frequency of all possible k-mers within mRNA sequences classified based on transcript region and localization. For each k-mer length, this analysis was performed for the group of top 40 HDLBP crosslinked k-mers in the membrane-bound CDS vs. cytosolic CDS (top) and cytosolic CDS vs. cytosolic 3'UTR (bottom).

(d) Distribution of z-scores calculated from differences in the frequency of all possible k-mers within mRNA sequences classified based on transcript region and localization. For each k-mer length, this analysis was performed for the group of top 40 HDLBP crosslinked k-mers in the membrane-bound CDS vs. cytosolic 3'UTR.

(e) P-values of two-sided Wilcoxon rank sum test between z-scores obtained for top 40 bound HDLBP k-mers as described in (D) and (E).

(f) Frequency of top 10 HDLBP crosslinked 4-mers located either in 3'UTR or CDS of membrane-bound and cytosolic mRNAs (left). Frequency of top 10 HDLBP crosslinked 4-mers located either in +40/-40 nt regions around crosslinks in 3'UTR or CDS of membrane-bound and cytosolic mRNAs (right).

(g) SDS-PAGE analysis of purified recombinant full-length (FL) and truncated HDLBP constructs (A-D) used in fluorescence polarization RNA binding assays.

(a - f) Source data are provided as a Source Data file.

Supplementary Figure 4

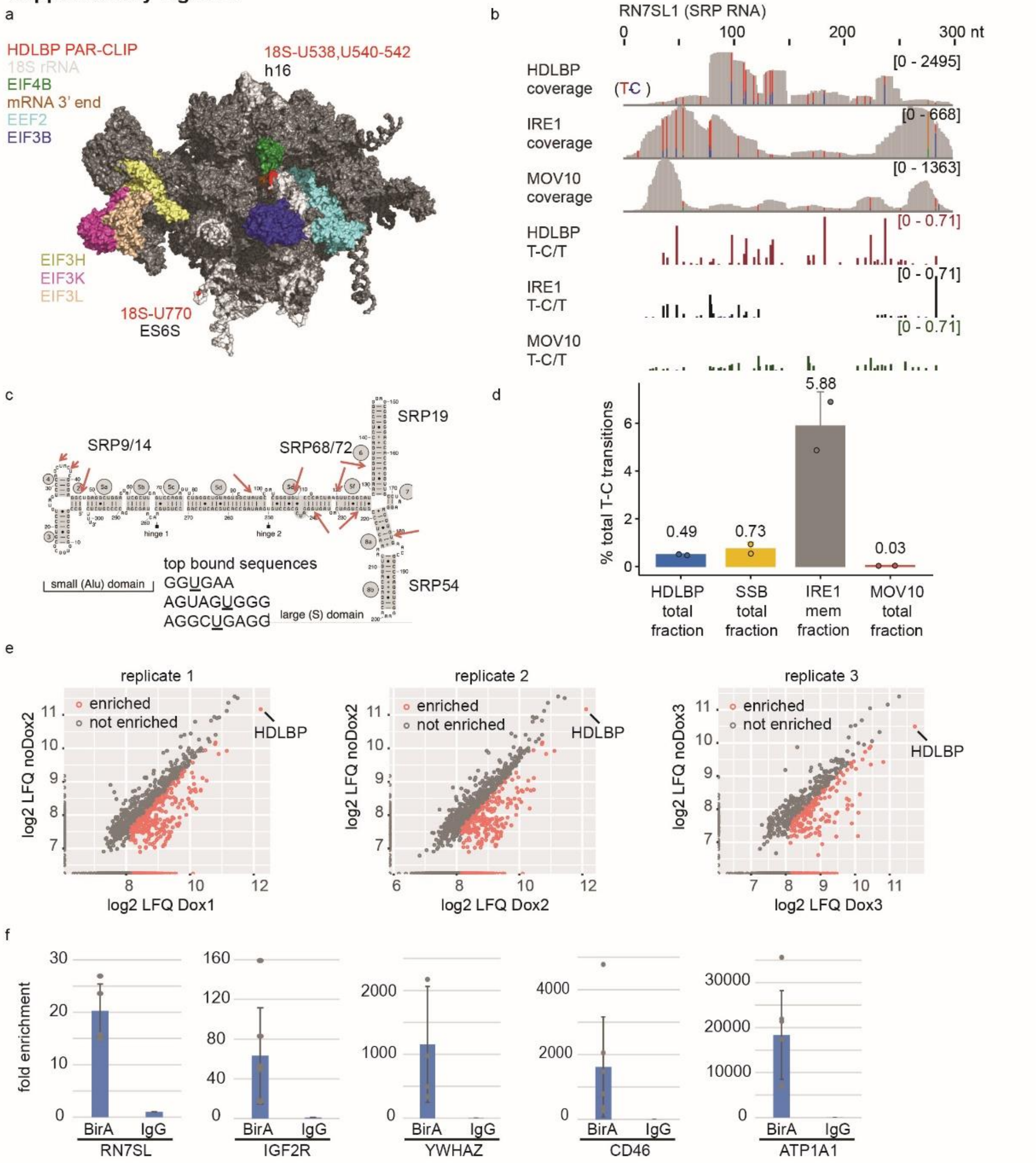

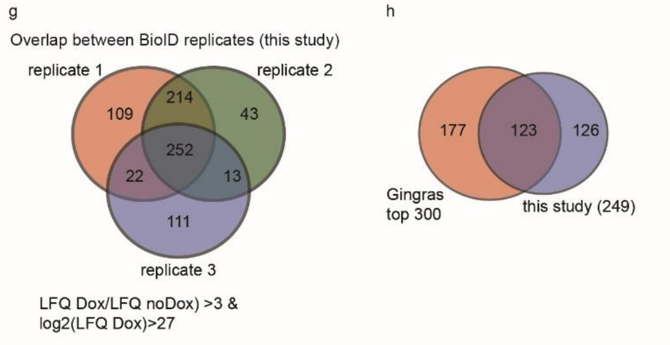

i

Translation

EIF4B FMR1 EIF3B EIF3A HBS1L FXR1 CNOT1YARSWARSDRG1EIF3IRPS10 RPS3A UPF1 EIF5 EIF4G2 EIF4G1 SYNCRIPYTHDF1EIF4ESTAT3YTHDF2 GIGYF2 DHX29 IGF2BP2 GEMIN5 TNRC6B EEF1D LARP4B XRN1 EEF1B2 EIF5A PUM1 CAPRIN1 EIF4E2 IGF2BP3 EIF2A LARP1 LARP4 PA2G4 LSM14A PPP1CA DDX1 ATXN2 PABPC4 PASK NACA

Supplementary Figure 4. HDLBP crosslinks to 18S rRNA and interacts with translation machinery

(a) Structures of the human 80S ribosome (PDB: 4V6X) and 48S pre-initiation complex (PDB: 6FEC) were juxtaposed and major HDLBP rRNA crosslinked nucleotides mapped (indicated in red).

(b) PAR-CLIP coverage and crosslinks on 7SL (SRP) RNA. For comparison, results for IRE1 and MOV10 PAR-CLIPs are included.

(c) Schematic of HDLBP PAR-CLIP crosslinks on the secondary structure of 7SL (SRP) RNA. Red arrows indicate crosslinked nucleotides. Approximate locations of SRP proteins are indicated.

(d) Percentage of total T-C transitions in the PAR-CLIP libraries is given for HDLBP, and for SSB, IRE1, MOV10 for comparison. Mean of 2 independent experiments (n=2) +/- SD was plotted for each protein.

(e) Scatter plots of log2-transformed LFQ intensity in doxycycline (Dox)-induced vs. uninduced replicate experiments. Enriched proteins (LFQ Dox/LFQ noDox>3 and log2(LFQ Dox)>27) are indicated as read dots, and the rest indicated in grey.

(f) RNA immunoprecipitation was performed using anti-FLAG immunoprecipitation with BirA*-FLAG-HDLBP as bait. Co-precipitating RNAs were quantified by qRT-PCR. Average fold enrichment (anti-FLAG vs. IgG control) from 4 replicates was calculated with error bars indicating standard deviation. Results are shown for 7SL RNA, IGF2R, YWHAZ, CD46 and ATP1A1.

(g) Reproducibility of the BirA*-FLAG-HDLBP BioID experiment. Venn diagram shows the overlap between enriched proteins in 3 independent biological replicate experiments. Absolute numbers are shown.

(h) Overlap between enriched proteins detected in this study (N-terminal BirA*, n=249) and published data (C-terminal BirA*)[44]. Absolute numbers are shown. Published dataset was ranked by the provided AvgP values (average probability across replicates).

(i) BioID identified proteins corresponding to the top enriched category (Translation) as determined by Gene Ontology enrichment analysis (Fig. 4e) are shown.

(a – h) Source data are provided as a Source Data file.

Supplemenraty Figure 5

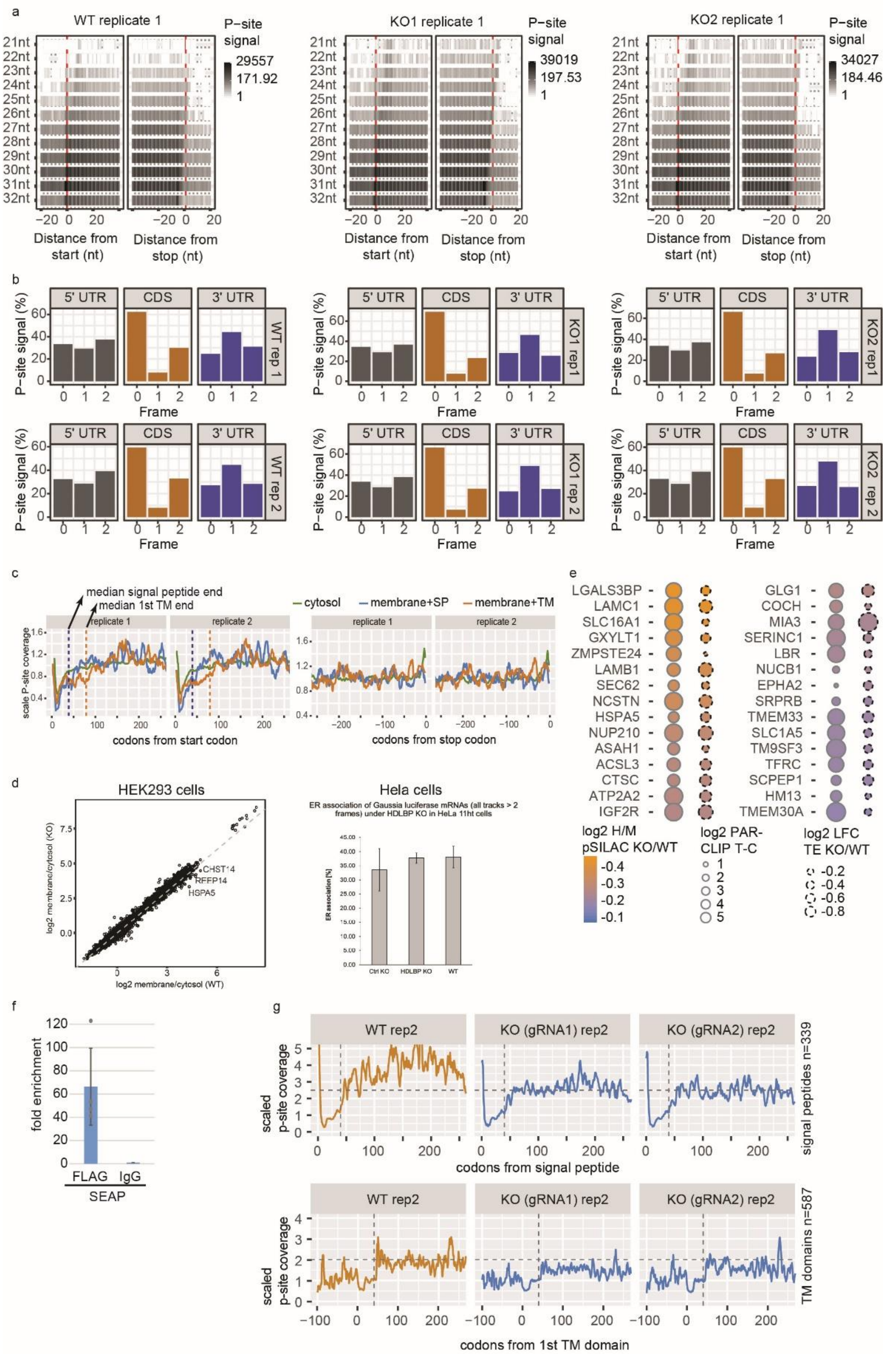

Supplementary Figure 5. HDLBP knockout reduces translation

(a) Quality control of ribosome profiling data and analysis of trinucleotide periodicity. Heatmaps show read coverage around start and stop codons for reads 21-32 nt in length.

(b) Quality control of ribosome profiling data and analysis of in-frame footprints. Bar plots show the percentages of P-sites detected in all three possible frames within transcript regions.

(c) P-site coverage was scaled to the mean P-site signal per CDS (total CDS coverage was required to be at least 5). For all interrogated codon positions within the CDS, the scaled P-site coverage was averaged over all CDS's. A rolling mean over 10 nt was used for smoothing.

(d) (Left) Scatter plot of log2-transformed membrane to cytosol RNA-seq read count ratios in fractionated HEK293 KO vs. WT cells (left panel). Depletion of HDLBP resulted in no apparent changes in mRNA localization (all DESeq P_{adj} >0.1). (Right) Co-localization of *Gaussia* luciferase mRNA with the ER was evaluated in HDLBP KO and WT HeLa cells using live cell imaging. Bar plots of percentage of ER localized *Gaussia* mRNA are shown. Error bars indicate standard error of mean. No significant differences between conditions were observed.

(e) Proteins with most decreased synthesis (top 30) after HDLBP KO as detected by pSILAC. Corresponding PAR-CLIP T-C signal and decrease in translational efficiency upon KO as detected by riboseq is shown.

(f) RNA immunoprecipitation was performed using anti-FLAG immunoprecipitation with FLAG/HA-HDLBP as bait in cells transfected with SEAP reporter. Co-precipitating SEAP RNAs were quantified by qRT-PCR. Average fold enrichment (anti-FLAG vs. IgG control) from 4 replicates was calculated with error bars indicating standard deviation.

(g) Ribosome P-site coverage around targeting signals (signal peptides and transmembrane helices) was compared between HDLBP KO vs. WT cells (replicate 2, compare with Fig. 5g for replicate 1). P-site coverage was scaled to the coverage in codons 20-40 of each mRNA. Rolling mean of 5 nt was used to smooth the profiles. Absolute numbers of analyzed mRNAs are given, median signal peptide and first transmembrane helix lengths are indicated with a vertical dotted line.

(a – g) Source data are provided as a Source Data file.

Supplementary Figure 6

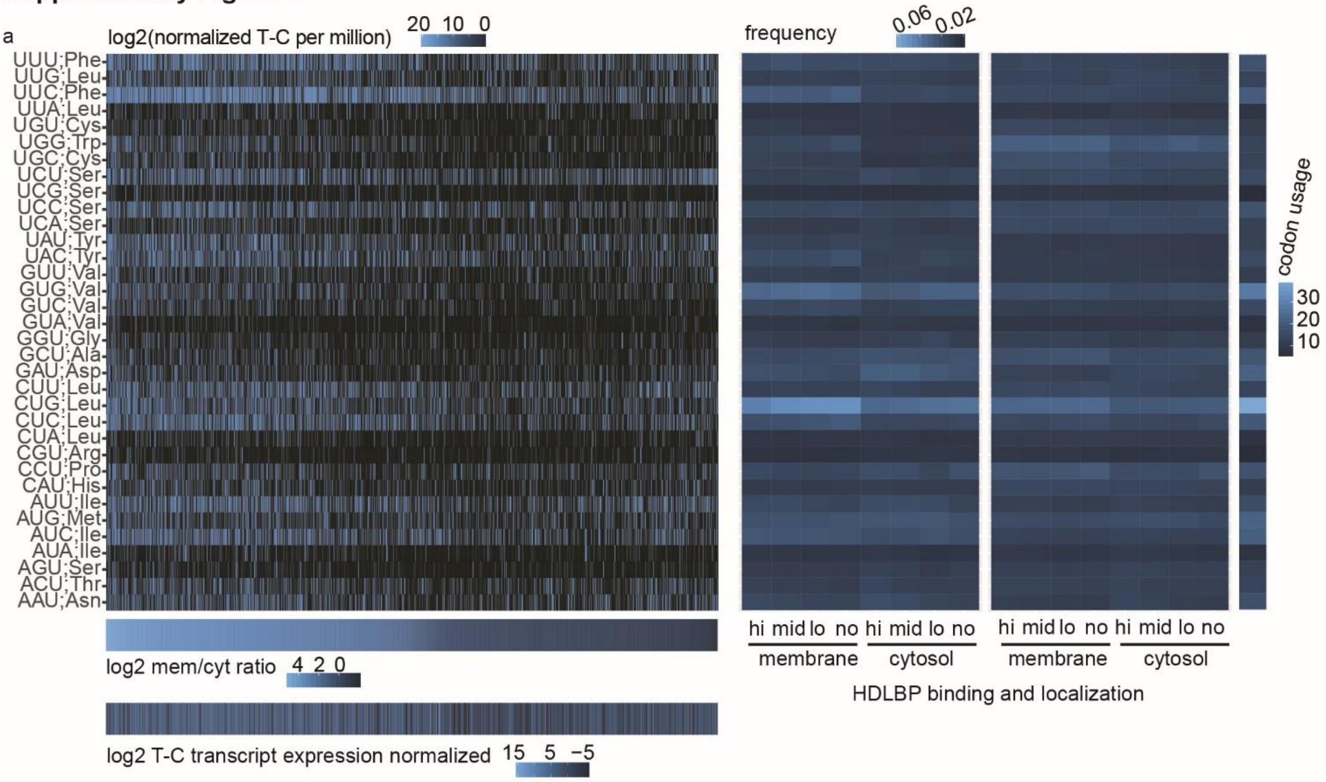

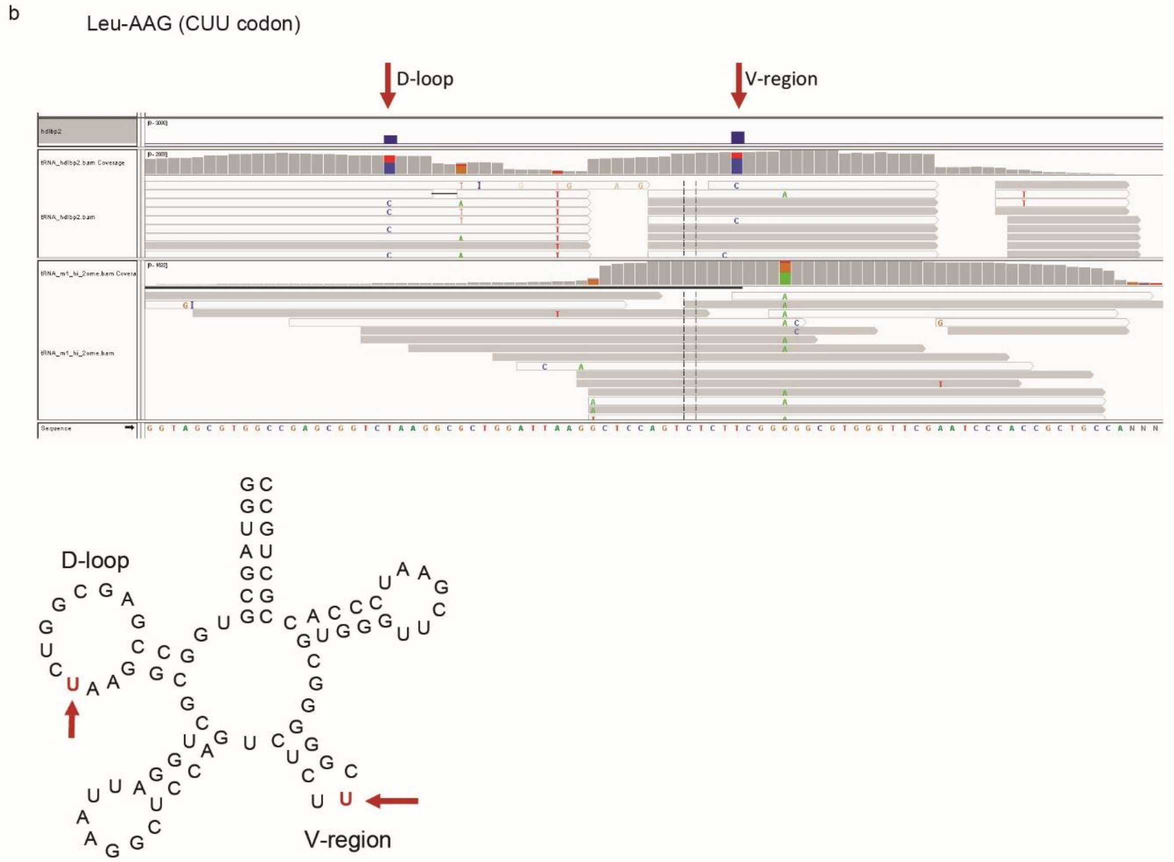

Supplementary Figure 6. HDLBP interacts with CU-rich codons in CDS and tRNAs

(a) (Left) Normalized T-C transitions were counted for each codon per transcript and normalized to the transcript expression level. Heatmaps shows transcripts (columns) ranked according to their membrane enrichment as determined by RNA-seq. (Middle) Panels show codon frequency in different classes according to mRNA localization and HDLBP PAR-CLIP binding. (Right) Panels show 3-mer frequency in different classes according to mRNA localization and HDLBP PAR-CLIP binding.

(b) (Top) Browser representation of alignment to tRNA Leu-AAG. T-C transitions in the D-loop and V-region are indicated for the HDLBP PAR-CLIP dataset. Second track shows coverage in the total RNA sample. (Bottom) HDLBP crosslinked uridines are indicated with respect to secondary tRNA structure.

(a, b) Source data are provided as a Source Data file.

Supplementary Figure 7

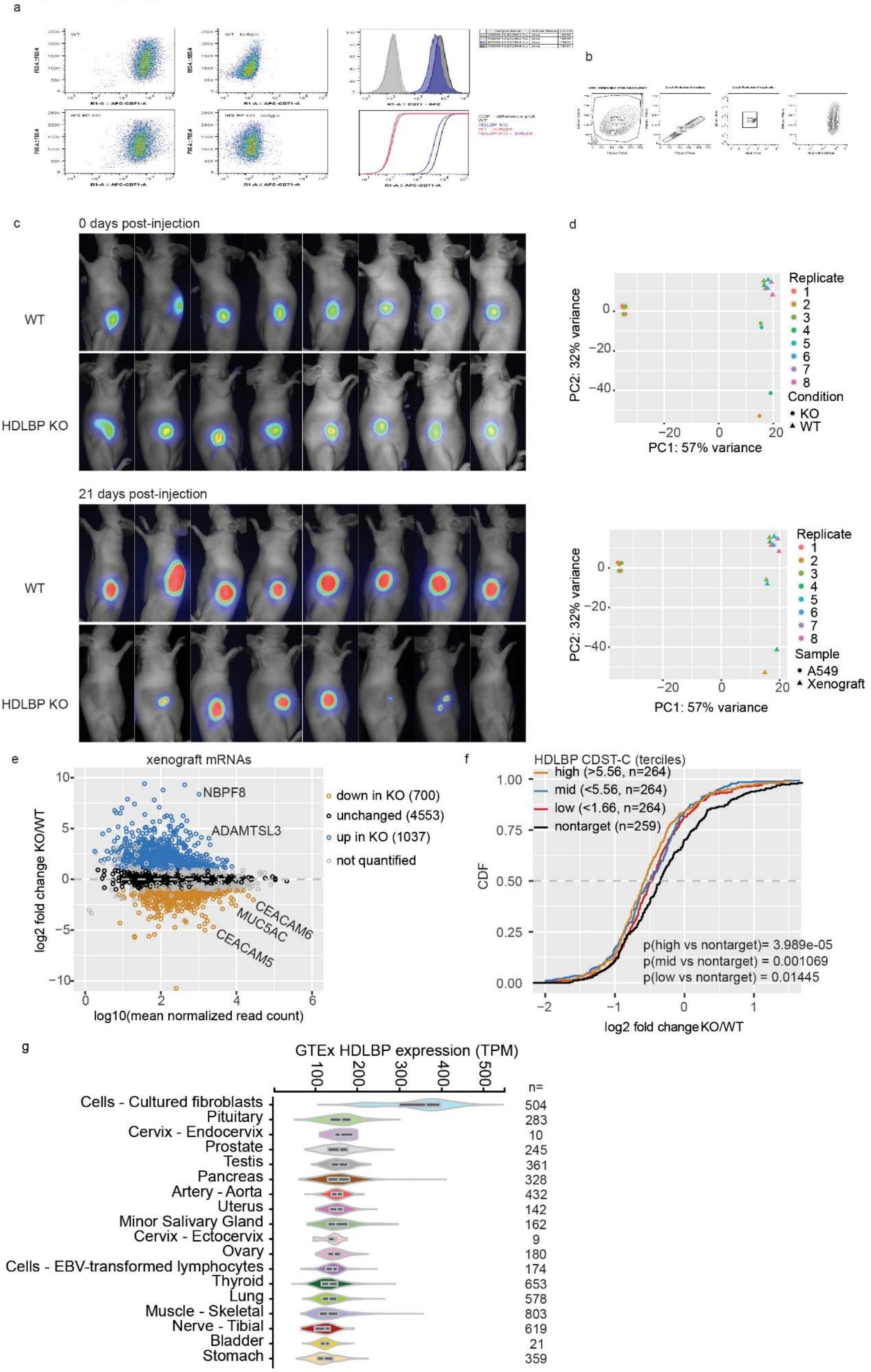

Supplementary Figure 7. Reduced tumor formation capacity of A549 HDLBP knockout cells

(a) Flow cytometry acquired pseudo-color plots of A549 WT and A549 HDLBP KO cells with CD71-APC and isotype-control staining. The mean fluorescent intensity of R1::CD71-APC was determined and is shown as histogram and CDF plot.

(b) Gating strategy for flow cytometry experiments presented in (a)

(c) Non-invasive infrared RFP imaging of mice 0 days post-injection confirming that the same amount WT and HDLBP KO cells were injected (upper panel) and 21 days post-injection showing the tumors (lower panel).

(d) Principal component analysis using variance stabilized transformation of RNA-seq read counts per gene. Shape of data points denotes either WT or HDLBP KO conditions (upper panel) or sample type (A549 cell line or xenograft tumors, lower panel). Colours denote biological replicates.

(e) Differential mRNA expression analysis (MA plot) between HDLBP KO and WT xenograft tumors. Log2-transformed fold changes (KO vs. WT) were plotted against log10-transformed mean normalized read count per mRNA. Upregulated mRNAs (log2 fold change >1 and P_{adj}<0.1) are indicated in blue and downregulated mRNAs in orange (log2 fold change<(-1) and P_{adj}<0.1). Unchanged mRNAs (log2 fold change <1 and log2 fold change >(-1) and P_{adj} >0.1) are shown in black.

(f) Differences in fold changes (HDLBP KO vs. WT tumors) were compared between different groups of mRNAs, defined according to HEK293 RNA-seq fractionation and PAR-CLIP experiments. Four similarly sized groups were selected based on HDLBP crosslinking signal in the CDS. Numbers of mRNAs per group are given in parentheses. Two-sided Wilcoxon rank-sum tests were used to test for significance.

(g) HDLBP mRNA expression in different tissues and cells according to GTEx (www.gtexportal.org). Top 18 tissues sorted by median HDLBP expression are shown. Number of samples for each tissue is indicated on the right. Lower and upper hinges of box plots correspond to the 25th and 75th percentiles, respectively. Upper and lower whiskers extend from the hinge to the largest or smallest value no further than 1.5 * interquartile range from the hinge, respectively. Center lines of box plots depict the median values.

(d – g) Source data are provided as a Source Data file.

Supplementary Table 1. Oligonucleotides

**Oligonucleotides**	
Cloning	
HDLBP_fwd_SphI	CGGCATGCAGTTCCGTTGCAGTTTTGACC
HDLBP_end_rev_NotI	ATAGTTTAGCGGCCGCCTATTATCGTTTGGGGCCCCA
HDLBP_A_fwd_BamHI	CGGGATCCAGTTCCGTTGCAGTTTTGACC
HDLBP_A_rev_NotI	ATAGTTTAGCGGCCGCCTACCGGTTAATCAAATCTTTGACC
HDLBP_B_fwd_SphI	CGGCATGCATGGACTATGTGGAGATCAACATCGAC
HDLBP_kh9_rev_NotI	ATAGTTTAGCGGCCGCCTAATTATCCAGGTTTTGGATCAAGG
HDLBP_C_fwd_BglII	GAAGATCTAACCTGGATAATGTGGTGGAAG
HDLBP_D_fwd_BamHI	CGGGATCCGATCTGGTGGAAAATAGCTATTCAATTTC
Gaussia_fwd	AACTGCTAGCATGGGAGTCAAAGTTCTGTTTG
Gaussia_rev	AATTGCGGCCGCTTAGTCACCACCG
SEAP_fwd	AACTGCTAGCATGCTGCTGCTGCTGCTGCTGCTGG
SEAP_rev	AATTGCGGCCGCCTAGCCGGCCGCCCCGACTCTAGA
qPCR	
ATP1A1_fwd	GGCAGTGTTTCAGGCTAACCAG
ATP1A1_rev	TCTCCTTCACGGAACCACAGCA
CD46_fwd	TGGCTACCTGTCTCAGATGACG
CD46_rev	GCATCTGATAACCAAACTCGTAAG
IGF2R_fwd	CGACTGCCAGTACCTCTTCT
IGF2R_rev	CGGGATTCTCGCTGTCAAAG
MT-CO1_fwd	GACGTAGACACACGAGCATATTTCA
MT-CO1_rev	AGGACATAGTGGAAGTGAGCTACAAC
RN7SL_fwd	TCAATATGGTGACCTCCCGG
RN7SL_rev	TTTTGACCTGCTCCGTTTCC
SEAP_fwd	TCAAGGGCAACTTCCAGAC
SEAP_rev	TTACCACTCCCACTGACTTC
YWHAZ_fwd	ACCGTTACTTGGCTGAGGTTGC
YWHAZ_rev	CCCAGTCTGATAGGATGTGTTGG
Ribosome profiling	
3’ adaptor 4N-RA3	rApp-NNNNTGGAATTCTCGGGTGCCAAGG-InvdT
5’ adaptor OR5-4N	rGrUrUrCrArGrArGrUrUrCrUrArCrArGrUrCrCrGrArCrGrArUrCrNrNrNrN
Marker-27 nt	rArUrGrUrArCrArCrGrGrArGrUrCrGrArGrCrUrCrArArCrCrCrGrC-P
Marker-30 nt	rArUrGrUrArCrArCrGrGrArGrUrCrGrArGrCrUrCrArArCrCrCrGrCrArArC-P
RT primer RTP	GCCTTGGCACCCGAGAATTCCA
PAR-CLIP	
3’adaptor 4N-RA3	rApp-NNNNTGGAATTCTCGGGTGCCAAGG-InvdT
5’ adaptor OR5-4N	rGrUrUrCrArGrArGrUrUrCrUrArCrArGrUrCrCrGrArCrGrArUrCrNrNrNrN

RT primer RTP	GCCTTGGCACCCGAGAATTCCA
ncRNA-seq	
3’adaptor 4N-RA3	rApp-NNNNTGGAATTCTCGGGTGCCAAGG-InvdT
5’adaptor 4N-SRC-cDNA	/5Phos/NNNNGATCGTCGGACTGTAGAACTCTGAAC-SpC3
RT primer RTP	GCCTTGGCACCCGAGAATTCCA
