# Supplementary material for: HDLBP binds ER-targeted mRNAs by multivalent interactions to promote protein synthesis of transmembrane and secreted proteins
Source: Nat Commun. 2022 May 18;13:2727. doi: 10.1038/s41467-022-30322-7 (PMC9117268; doi:10.1038/s41467-022-30322-7)
Supplement: Supplementary file 3 — Description of Additional Supplementary Files [file 41467_2022_30322_MOESM3_ESM.pdf]

Description of Additional Supplementary Files

File Name: Supplementary Data 1
Description: Membrane-to-cytosol enrichment as determined by RNA-seq including the determined localization category (membrane or cytosol), expression level (tpm_wt), enrichment (log2 fold change) and DESeq2-derived P values adjusted for multiple comparisons. Wald test was used for comparisons.

File Name: Supplementary Data 2
Description: HDLBP PAR-CLIP signal per transcript including number of T-C transitions, uniquely mapping reads or number of clusters per compartment (5‟UTR, CDS, 3‟UTR or whole transcript)

File Name: Supplementary Data 3
Description: HDLBP PAR-CLIP clusters per replicate (1, 2) including chromosome coordinates (hg19) per cluster, number of T-C transitions, number of uniquely mapping reads per cluster, number of overlapping nucleotides between clusters, and the corresponding transcript region (5‟UTR, CDS, 3‟UTR).

File Name: Supplementary Data 4
Description: BioID results table including LFQ values and calculated enrichment per replicate (1-3), as well as mean enrichment and mean LFQ values.

File Name: Supplementary Data 5
Description: Differences in translation efficiency (TE - log2 fold change ribo rna) between wild-type (293) and knockout conditions (guide 1 or 2), as well as mean wild-type vs. knockout TE and DESeq2-derived P values adjusted for multiple comparisons. Likelihood ratio test was used for comparisons.

File Name: Supplementary Data 6
Description: pSILAC results table including iBAQ values normalized SILAC heavy-tomedium (H/M) ratios per replicate (1, 2), mean H/M and per protein information with respect to the corresponding mRNA localization, targeting signal presence and HDLBP target category

File Name: Supplementary Data 7
Description: HDLBP PAR-CLIP tRNA enrichment table including T-C transitions in tRNA coordinates (tc_start, tc_stop), number of T-C transitions and total read coverage for every position in both replicates (TCnumber_hdlbp2 TCnumber_hdlbp3, allReadsPerPos_hdlbp2 allReadsPerPos_hdlbp3), sequence of the crosslink-centred 7-mer (seq_tc_middle_t), normalized number of T-C transitions, mean fraction of T-C transitions over total coverage, standard deviation of fraction of T-C transitions over total coverage, T-C transition signal in whole tRNA normalized to the total tRNA abundance

File Name: Supplementary Data 8
Description: Xenograft tumor RNA-seq results table with upregulated and downregulated mRNAs in the HDLBP KO condition including mean of normalized read counts (baseMean), log2 fold changes, DESeq2-derived Padj values and TPM values for replicate experiments.
