# Supplementary material for: HDLBP binds ER-targeted mRNAs by multivalent interactions to promote protein synthesis of transmembrane and secreted proteins
Source: Nat Commun. 2022 May 18;13:2727. doi: 10.1038/s41467-022-30322-7 (PMC9117268; doi:10.1038/s41467-022-30322-7)
Supplement: Supplementary file 12 — Reporting Summary [file 41467_2022_30322_MOESM12_ESM.pdf]

nature research

Corresponding author(s): Miha Milek, Markus Landthaler

Last updated by author(s): Mar 16, 2022

Reporting Summary

Nature Research wishes to improve the reproducibility of the work that we publish. This form provides structure for consistency and transparency in reporting. For further information on Nature Research policies, see our Editorial Policies and the Editorial Policy Checklist.

Statistics

For all statistical analyses, confirm that the following items are present in the figure legend, table legend, main text, or Methods section.

n/a	Confirmed	
☐	☒	The exact sample size (*n*) for each experimental group/condition, given as a discrete number and unit of measurement
☐	☒	A statement on whether measurements were taken from distinct samples or whether the same sample was measured repeatedly
☐	☒	The statistical test(s) used AND whether they are one- or two-sided<br>*Only common tests should be described solely by name; describe more complex techniques in the Methods section.*
☐	☒	A description of all covariates tested
☐	☒	A description of any assumptions or corrections, such as tests of normality and adjustment for multiple comparisons
☐	☒	A full description of the statistical parameters including central tendency (e.g. means) or other basic estimates (e.g. regression coefficient) AND variation (e.g. standard deviation) or associated estimates of uncertainty (e.g. confidence intervals)
☐	☒	For null hypothesis testing, the test statistic (e.g. *F*, *t*, *r*) with confidence intervals, effect sizes, degrees of freedom and *P* value noted<br>*Give P values as exact values whenever suitable.*
☒	☐	For Bayesian analysis, information on the choice of priors and Markov chain Monte Carlo settings
☒	☐	For hierarchical and complex designs, identification of the appropriate level for tests and full reporting of outcomes
☐	☒	Estimates of effect sizes (e.g. Cohen's *d*, Pearson's *r*), indicating how they were calculated

Our web collection on statistics for biologists contains articles on many of the points above.

Software and code

Policy information about availability of computer code

Data collection	For high-throughput sequencing data collection we used Illumina HiSeq 2500, 4000 and NextSeq 500 platforms. Basecalls were converted to fastq files using Bcl2Fastq (version 2.16.0.10). Data collection for BioID and pSILAC proteomics experiments was performed on LC-MS/MS system (EkspertNanoLC 415 [Eksigent] and on Orbitrap HF-X/OrbitrapExploris 480 mass spectrometers, respectively. Mice were imaged for iRFP using a Pearl Trilogy Imaging System (LI-COR). The fluorescence intensity was determined by using the Image Studio Lite (v5.2.5) software (LI-COR).
Data analysis	- High-throughput sequencing analysis:<br>Reads were demultiplexed and adapter sequences removed by Flexbar (2.5). Ribo-seq and PAR-CLIP reads were then collapsed to remove PCR duplicates, followed by removal of random nucleotides (four on both 5' and 3' end of the reads) using fastx_trimmer (FASTX Toolkit 0.0.14). Reads aligning to rRNA sequences and other sources of contamination using a custom index were removed by Bowtie2 (v2.3.2) and the remaining sequences were aligned to the human transcriptome (hg19) using STAR aligner (2.5.3a) using GTF annotation file Gencode v19. Transcriptome BAM files were converted to the bed format using BedTools bamToBed (v2.26.0). Bed files were then input into riboWaltz (v1.1.0) (PMID:30102689).<br>For Ribo-seq translation efficiencies per gene we used RSEM (v1.2.2) which supplied us with read counts and TPM values per gene. Differences in translational efficacy, as well as in mRNA abundance and due to both effects (transcription and translation) were detected by DESeq2 (1.18.1) with an interaction term model as described previously (PMID: 31763789).<br>PAR-CLIP reads were aligned to the human genome (hg19 build) using BWA v0.7.15-r1140 by using the previously published computational PAR-CLIP pipeline (v0.97a) (Lebedeva et al. 2011; Jens 2016). https://github.com/marvin-jens/clip_analysis.<br>RNA-seq read counts per gene and TPM values were obtained by RSEM (v.1.2.20) (Li and Dewey, 2011) using default parameters and Bowtie (v.1.1.2) (Langmead et al., 2009) as transcriptome alignment program.<br>For tRNA quantification from PAR-CLIP and ncRNA-seq reads we used a tRNA reference genome that contained 58 unique tRNAs. Mapping was performed using Bowtie2 (v.2.3.2) and custom scripts using BedTools (v2.26.0) intersect and getfasta commands were used to obtain T-C transition counts per position and per tRNA. We excluded tRNA dihydroridine positions using BedTools. The tRNA sequences, dihydrouridine positions along with the entire tRNA processing pipeline are available from GitHub https://github.com/mmilek/hdlbp/trna/.

- pSILAC:
Raw files were analyzed together with MaxQuant software (v1.6.0.1) with default parameters.
- BioID: MaxQuant proteomics pipeline (v1.5.3.30 and v1.5.8.3) was used for raw data analysis and the Andromeda search engine (PMID: 21254760),
- All additional analysis was performed in R environment (v3.6.1). The analysis scripts and processed data are publicly available from GitHub at https://github.com/mmilek/hdlbp.git.

For manuscripts utilizing custom algorithms or software that are central to the research but not yet described in published literature, software must be made available to editors and reviewers. We strongly encourage code deposition in a community repository (e.g. GitHub). See the Nature Research guidelines for submitting code & software for further information.

Data

Policy information about availability of data

All manuscripts must include a data availability statement. This statement should provide the following information, where applicable:

- Accession codes, unique identifiers, or web links for publicly available datasets
- A list of figures that have associated raw data
- A description of any restrictions on data availability

PAR-CLIP, RNA-seq and ribosome profiling data from this study have been submitted to the NCBI Gene Expression Omnibus (GEO; http://www.ncbi.nlm.nih.gov/geo/) under accession GSE148262. BioID and pSILAC data have been submitted to the ProteomeXchange (http://www.proteomexchange.org) under the data set identifiers PXD018313 and PXD018316 respectively. All analysis scripts and processed data are publicly available from GitHub at https://github.com/mmilek/hdlbp_rev.git . Source data are provided as a Source Data file.
List of figures that have associated raw data:
Figure 1: RNA-seq and PAR-CLIP
Figure 2: PAR-CLIP
Figure 3: PAR-CLIP
Figure 4: PAR-CLIP and BioID
Figure 5: PAR-CLIP, Ribo-seq and pSILAC
Figure 6: Ribo-seq and ncRNA-seq
Figure 7: RNA-seq
Figure S1: RNA-seq and PAR-CLIP
Figure S2: PAR-CLIP
Figure S3: PAR-CLIP
Figure S4: PAR-CLIP and BioID
Figure S5: Ribo-seq and pSILAC
Figure S6: Ribo-seq and ncRNA-seq
Figure S7: RNA-seq .

IRE1 PAR-CLIP92 was obtained by communication with the corresponding author, SSB PAR-CLIP datasets were obtained from GEO under accession “GSE95683" and from SRA under accession “SRR4301753". MOV10 PAR-CLIP was obtained from GEO under accession “GSE48245”.

Field-specific reporting

Please select the one below that is the best fit for your research. If you are not sure, read the appropriate sections before making your selection.

☒ Life sciences ☐ Behavioural & social sciences ☐ Ecological, evolutionary & environmental sciences

For a reference copy of the document with all sections, see nature.com/documents/nr-reporting-summary-flat.pdf

Life sciences study design

All studies must disclose on these points even when the disclosure is negative.

Sample size	No sample-size calculation was performed prior to the experimental work. Due to the fact that most high-throughput experiments required many conditions (subcellular fractions) and were thus laborious and costly, we minimized the number of biological replicates, while keeping the number of conditions high. For these experiments, we achieved high level of reproducibility for two or more biological replicates as indicated in the manuscript. In other low throughput follow-up experiments, we increased the number of biological replicates to three or four and assessed the level of reproducibility using standard deviation calculation. For tumor xenograft analysis up to eight replicates were performed as indicated in the manuscript.
Data exclusions	Generally, no data were excluded from the analysis. For some analyses of high-throughput sequencing or proteomics data, we set thresholds to exclude transcripts with insufficient coverage, or proteins with low label-free quantification values. This is a common strategy for high-throughput data analysis. The thresholds used are specified in Figure legends and Materials and Methods.
Replication	All attempts at replication were successful. For high-throughput sequencing experiments, we used two biological replicates per condition, for pSILAC experiments four and for BioID three biological replicates were used. In other low throughput experiments, we increased the number of biological replicates to three or four.
Randomization	Samples were allocated non-randomly to different experimental groups based on the different conditions used during cell culture based studies.

Blinding	Since not applicable, investigators were not blinded during data collection and analysis.

Reporting for specific materials, systems and methods

We require information from authors about some types of materials, experimental systems and methods used in many studies. Here, indicate whether each material, system or method listed is relevant to your study. If you are not sure if a list item applies to your research, read the appropriate section before selecting a response.

Materials & experimental systems

n/a	Involved in the study
☐	☒ Antibodies
☐	☒ Eukaryotic cell lines
☒	☐ Palaeontology and archaeology
☐	☒ Animals and other organisms
☒	☐ Human research participants
☒	☐ Clinical data
☒	☐ Dual use research of concern

Methods

n/a	Involved in the study
☒	☐ ChIP-seq
☐	☒ Flow cytometry
☒	☐ MRI-based neuroimaging

Antibodies

Antibodies used

Western Blot
anti-HDLBP (Abcam, ab109324), anti-beta-Tubulin (Sigma, T8328, clone AA2), anti-HA (Covance, MMS-101P-1000), anti-BCAP31 (Proteintech, 11200-1-AP), anti-RPS6 (Cell Signaling,#2217), anti-RPL7 (Abcam, ab72550), goat anti-rabbit immunoglobulins/HRP (Agilent cat# P044801-2), and goat anti-mouse immunoglobulins/HRP (Agilent cat# P044701-2)

Immunoprecipitation
anti-FLAG (Sigma, F1804)

FACS
anti-CD71 (Miltenyi Biotec, RRID:AB_2660542), REA Control Antibody (S), human IgG1 (Miltenyi Biotec, RRID:AB_2733447)

Validation

Antibodies were validated for species and application by manufacturers. Quality assessments for all antibodies used in this study are provided online by manufacturers.
anti-HDLBP (Abcam, ab109324) : Suitable for Western Blot(WB) and IHC-Ped.
anti-beta-Tubulin (Sigma, T8328) : Suitable for WB
anti-HA (Covance, MMS-101P-1000): Suitable for WB
anti-BCAP31 (Proteintech, 11200-1-AP): Suitable for WB
anti-RPS6 (Cell Signaling,#2217): Suitable for WB
anti-RPL7 (Abcam, ab72550) : Suitable for WB
anti-FLAG (Sigma, F1804): Suitable for immunoprecipitation
anti-CD71 (Miltenyi Biotec): Suitable for flowcytometry

Eukaryotic cell lines

Policy information about cell lines

Cell line source(s)	HEK 293 Flp-In TRex (Thermo Fischer Scientific Cat. No. R78007), A549 (ATCC Cat.No. CCL-185)
Authentication	Both cell lines were purchased directly from the manufacturers. The manufacturers authenticated them by certification. We kept both cell lines at low passage number but did not authenticate them ourselves.
Mycoplasma contamination	Cell lines used in this study were tested negative for mycoplasma contamination.
Commonly misidentified lines (See ICLAC register)	Both cell lines have been commonly misidentified according to ICLAC. The rationale behind using them is that HEK 293 Flp-In TRex allow inducibly controllable expression of proteins of interest from a defined genomic locus. A549 cells were chosen since these cells are an established model for xenograft experiments.

Animals and other organisms

Policy information about studies involving animals; ARRIVE guidelines recommended for reporting animal research

Laboratory animals

species: athymic nude mouse
strain: Crl:NU(NCr)-Foxn1nu
sex: female
number: 8
age: 6 weeks

Wild animals	The study did not involve wild animals.
Field-collected samples	The study did not involve samples collected from the field.
Ethics oversight	An ethical review committee of the Martin-Luther-University Halle-Wittenberg approved the xenograft assays.<br>Ethical approval: Landesverwaltungsamt Sachsen-Anhalt

Note that full information on the approval of the study protocol must also be provided in the manuscript.

Flow Cytometry

Plots

Confirm that:

☒ The axis labels state the marker and fluorochrome used (e.g. CD4-FITC).

☒ The axis scales are clearly visible. Include numbers along axes only for bottom left plot of group (a 'group' is an analysis of identical markers).

☒ All plots are contour plots with outliers or pseudocolor plots.

☒ A numerical value for number of cells or percentage (with statistics) is provided.

Methodology

Sample preparation	200000 HDLBP knockout and WT control cells were seeded in 6-well plates and grown for 2 days. After harvesting using trypsin, cells were counted. For CD71 surface labelling, 300000 cells were stained with anti-CD71 antibodies or isotype control diluted in 1% BSA/PBS for 15 min at 4°C. After washing with PBS, cells were analyzed by flow cytometry.
Instrument	MACSQuant Analyzer (Core Facility Imaging; Martin-Luther University Halle-Wittenberg)
Software	FlowJo
Cell population abundance	Both, WT and HDLBP-KO cells are 100% positive for CD71-APC. Expression changes are determined by alterations of the mean fluorescence intensities.
Gating strategy	Cells were identified by the FCS-A/SSC-A scatter plot. Doublets were excluded using the FSC-A/FSC-H scatter. Dead cells were determined by propidium iodide staining and excluded from the population using the B3::PI-A/B3::PI-H scatter. Changes in the median fluorescence intensities were determined and are shown as pseudocolor plots, histogram and CDF plot.

☒ Tick this box to confirm that a figure exemplifying the gating strategy is provided in the Supplementary Information.
